# Plasma Level of Human Epididymis Protein 4 and Risk of Recurrence after First Venous Thromboembolism: The HUNT Study

**DOI:** 10.1055/a-2961-2256

**Published:** 2026-09-30

**Authors:** Celina J. Cathro, Sigrid K. Brækkan, Kristian Hveem, John-Bjarne Hansen

**Affiliations:** 1 Department of Clinical Medicine 8016 Thrombosis Research Group (TREC), UiT The Arctic University of NorwayTromsøTromsNorway; 2 Division of Internal Medicine 60519 Thrombosis Research Center (TREC), University Hospital of North NorwayTromsøTromsNorway; 3 Department of Public Health and Nursing 8018 HUNT Center for Molecular and Clinical Epidemiology, Norwegian University of Science and TechnologyTrondheimTrøndelagNorway; 4 8018 HUNT Research Center, Norwegian University of Science and TechnologyTrondheimTrøndelagNorway; 5 Department of Research 60510 St Olav’s Hospital HFTrondheimTrøndelagNorway

**Keywords:** venous thrombosis, deep vein thrombosis, pulmonary embolism, recurrence risk, human epididymis protein 4, HUNT study

## Abstract

**Background** Patients with incident venous thromboembolism (VTE) have a high risk of recurrence. Recent studies have shown that plasma levels of human epididymis protein 4 (HE4) are associated with increased risk of future incident VTE.

**Objective** To investigate whether plasma HE4 levels are associated with risk of recurrent VTE.

**Methods** Patients with first-lifetime VTE ( *n* = 896) derived from the Trøndelag Health Study (HUNT3; 2006–2019) were followed from the VTE date. Symptomatic and objectively confirmed VTE recurrences were recorded during the study period. Plasma HE4 levels were measured by Somascan at the time of inclusion in the source cohort (HUNT3, prior to incident VTE). Cox regression models were used to estimate hazard ratios (HRs) for VTE recurrence across quartiles of plasma HE4 levels adjusted for age, sex, and body mass index. Subgroup analyses by type of incident VTE were also performed.

**Results** During follow-up, 116 patients developed recurrent VTE. We found no significant association between quartiles of plasma HE4 levels and absolute or relative risk of recurrent VTE. The HR for recurrence in individuals with elevated plasma HE4 levels (Q4) versus low HE4 levels (Q1) was HR: 0.82 (95% confidence interval [CI]: 0.46–1.49). Similarly, no association was found between plasma HE4 levels and recurrent VTE in any subgroup analyses (index deep vein thrombosis, pulmonary embolism, provoked VTE, or unprovoked VTE).

**Conclusion** Our results suggest that inherent plasma HE4 levels are not useful as a risk biomarker for recurrent VTE.

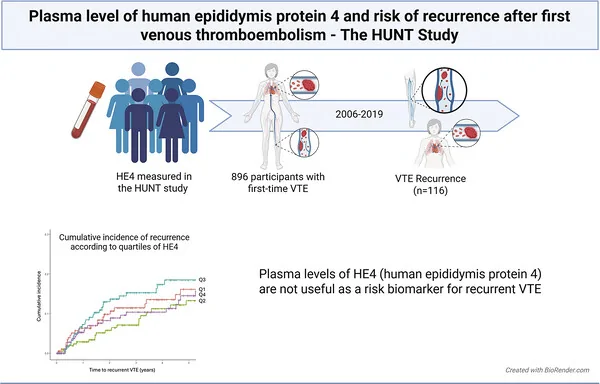

## Introduction

Venous thromboembolism (VTE), encompassing deep vein thrombosis (DVT) and pulmonary embolism (PE), 1 affects 1 in 12 individuals throughout lifetime, 2 accounting for approximately 10 million VTEs worldwide annually. 1 A substantial proportion of those who survive VTE experience long-term health complications, such as postthrombotic syndrome, 3 post-PE syndrome, 4 and recurrence, 5 6 psychosocial complications such as depression 7 and economic burdens such as disability pension. 8 Recurrent VTE occurs in approximately 30% of patients within 10 years of the incident diagnosis, of which most events occur within the first year. 5 6 Recurrent VTE is more costly and resource-demanding compared with the incident event 9 and further augments the risk of severe complications and death. 10 11 12 13 14

Several predictive biomarkers have been discovered, and risk assessment models (RAM) developed to identify VTE patients at risk of recurrence. 15 16 Even though these RAMs can to some extent discriminate VTE patients with high and low risk of recurrence at the population level, they are not recommended for use in international guidelines due to lack of discriminatory power at the individual level. 17 18 19 20 Extended anticoagulant treatment can efficiently reduce the risk of VTE recurrence, but at an added risk of major bleeding. 17 21 Thus, it is warranted to identify novel biomarkers of recurrent VTE that could improve risk stratification and thereby the benefit-to-harm ratio for individual VTE patients when offering extended anticoagulant treatment.

Human epididymis protein 4 (HE4), belonging to the Whey Acidic Protein Four-Disulfide Core domain family, 22 functions as a protease inhibitor and is widely recognized as a biomarker for cancer diagnosis and progression, 23 24 25 most notably ovarian cancer. 26 27 Additionally, HE4 levels have been identified as a prognostic biomarker of heart failure severity 28 and recurrence of atrial fibrillation after catheter ablation. 29 Moreover, HE4 levels are elevated in patients with COPD, 30 cardiovascular complications in COPD, 31 and chronic kidney disease. 32

HE4 has previously been reported to be associated with VTE in selected patient populations, 33 34 and recently we demonstrated a linear relationship between plasma HE4 levels and risk of future incident VTE in a case-cohort derived from the general population. 35 Notably, the association was not explained by cancer or cancer-related VTE. 35 Individuals with elevated HE4 levels (highest quartile) had a 2.5-fold higher risk of incident VTE than individuals with low HE4 levels (lowest quartile). 35 However, no previous study has investigated the association between plasma HE4 levels and risk of recurrent VTE. Therefore, we aimed to investigate whether plasma HE4 levels, measured prior to the incident VTE, were associated with risk of recurrent VTE in a cohort of incident VTE patients recruited from the general population.

## Methods

### Study Population and Design

Our study population was derived from the third survey of the Trøndelag Health Study (HUNT3), a population-based cohort (survey inclusion in 2006–2008) that invited all inhabitants aged 20 and older to participate. 36 37 The participation rate was 51.4% ( *n* = 50,800), and all study participants provided written informed consent prior to inclusion. Participants were excluded from the source cohort if they had been diagnosed with VTE ( *n* = 622), abdominal aortic aneurysm ( *n* = 100, due to a related project), cancer ( *n* = 1908), or had missing plasma samples ( *n* = 1657) prior to inclusion. After application of the exclusion criteria, 46,513 eligible participants remained in the source cohort. Baseline information of HUNT3 participants was collected at inclusion by self-reported questionnaires, clinical examinations, and collection of blood samples. Nonfasting blood samples were collected into vacutainer tubes containing ethylenediaminetetraacetic acid as an anticoagulant, mixed, and centrifuged for 10 minutes at 2500 g (6 °C). Plasma samples were stored at −80 °C in the HUNT Biobank. 38 Information on arterial cardiovascular disease (CVD) history (myocardial infarction, stroke, and/or angina pectoris; yes/no) and daily smoking (yes/no) were extracted from the self-reported questionnaires. Body height and weight were objectively measured, and body mass index (BMI) was calculated as participant weight in kilograms divided by height in square meters (kg/m ^2^ ).

This study utilized a cohort design including all participants from HUNT3 who experienced an incident VTE from inclusion until the end of follow-up (December 31, 2019). Amongst the eligible participants, 911 experienced an incident VTE, and 15 participants were excluded due to death on the same day as the incident VTE. Therefore, the final study population consisted of 896 participants. A flowchart of the study inclusion is shown in Fig. 1 .

**Fig. 1:**
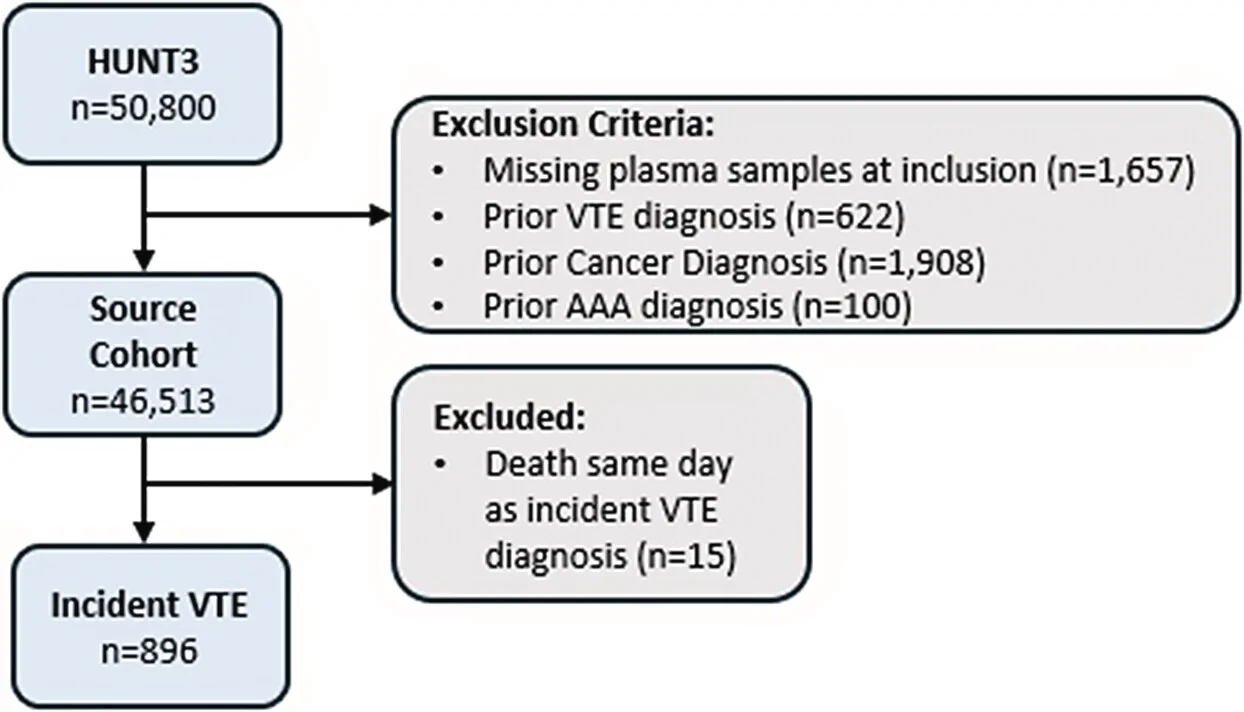
Participant flowchart. AAA, abdominal aortic aneurysm; VTE, venous thromboembolism.

### Registration and Validation of Incident and Recurrent VTE Events

The incident VTE events were identified by searching the autopsy and discharge diagnosis registries at the three included hospitals (Namsos Hospital, Levanger Hospital, and St. Olav’s Hospital, Trondheim) in the catchment area of HUNT using the relevant ICD-10 codes. 35 After review of participants’ medical records by trained medical professionals, potential VTE cases were confirmed if signs and symptoms of DVT and/or PE were recorded, radiologically confirmed, leading to a diagnosis, and the patient received treatment (except in the case of contraindications). In addition, medical records for the 3 months prior to VTE diagnosis were reviewed, and information regarding provoking and/or risk factors was extracted using a standardized form. If a participant experienced both DVT and PE, the event was classified as a PE. A VTE was classified as provoked if it occurred within 3 months following any of these provoking factors: active cancer, surgery, immobilization (wheelchair confinement, plaster cast, or bed rest for at least 3 days), trauma, acute medical conditions (stroke, myocardial infarction, or severe infection), or any other factor(s) recorded as provoking on participant medical records (e.g., intravascular catheter). If the provoking factor was active cancer, the VTE was classified as cancer-associated thrombosis. If none of the above provoking factors were present, the VTE was classified as unprovoked.

Any new symptomatic VTE event occurring after the incident VTE during the follow-up period was included as a recurrent VTE. Recurrent VTE events were identified and objectively validated using the same methods as described above for incident VTE events.

### Plasma HE4 Measurements

Plasma samples collected at inclusion in HUNT3 were sent from the HUNT biobank 38 to the Somalogic Laboratories in Boulder, Colorado, United States, for semiquantitative measurement of HE4 using the Somascan 7k aptamer-based platform according to standard protocol. 39 The coefficient of variation (CV) reported by Somalogic for HE4 in human plasma is 5.3%. 40

### Statistical Analysis

Statistical analyses were computed using R Version 4.3.1 (The R Foundation for Statistical Computing, Vienna, Austria). Raw plasma HE4 measurements from the Somascan data were log2-transformed and normalized so that the mean and standard deviation (SD) were equal to 0 and 1, respectively. Participants were divided into quartiles of plasma HE4 levels based on quartile cutoffs derived from the subcohort when investigating the association between HE4 levels and risk of future incident VTE. 35 These quartile cutoffs were used as we observed a higher risk of incident VTE in those with higher HE4 levels, and as clinical values for HE4 in relation to VTE have yet to be determined. Descriptive statistics were performed to display baseline characteristics of the study population. Continuous variables were presented as median with interquartile range or as mean ± SD, while categorical variables were presented as percentages with numbers in brackets.

For each participant, person-time of follow-up was calculated from the date of incident VTE until the date of recurrence, death, or end of follow-up (December 31, 2019), whichever happened first. Incidence rates (IR) and 95% confidence intervals (CI) for recurrent VTE were calculated and expressed per 100 person-years (PY) according to quartiles (Q1–Q4) of plasma HE4 levels. Furthermore, we applied Cox proportional hazards regression models to estimate hazard ratios (HR) and 95% CIs for recurrent VTE according to quartiles of plasma HE4 levels. The first analysis (model 1) was adjusted for age, sex, and sample batch, while the second analysis (model 2) was further adjusted for BMI. All analyses were repeated for subgroups restricted to the type of index VTE (i.e., DVT, PE, provoked VTE, and unprovoked VTE). To evaluate potential nonlinear association between HE4 and recurrence, we additionally applied a cubic spline plot with 5 knots entering HE4 (log2-transformed and standardized to a mean of 0 and SD of 1) as a continuous variable.

Unfortunately, we did not have access to information regarding anticoagulation treatment following the incident VTE diagnosis. Therefore, we performed a sensitivity analysis where we started the follow-up time after the first 3 and 6 months following the index VTE. This was done to assess whether the duration of anticoagulation treatment could have impacted the results. In addition, we performed a second sensitivity analysis to investigate whether any potential fluctuation in plasma HE4 levels following inclusion into the HUNT3 study until incident VTE diagnosis could have impacted our results. To do this, we added the number of days from source cohort inclusion date until date of incident VTE diagnosis as a covariate (model 2 + time).

The 5-year cumulative incidence of overall VTE recurrence from the time of incident VTE according to quartiles of plasma HE4 levels was displayed in a 1-Kaplan–Meier plot.

## Results

The characteristics of participants at study inclusion across quartiles of plasma HE4 levels are displayed in Table 1 . The mean age, number of participants, percentage of daily smokers, and those with a history of CVD increased from Q1 to Q4. Conversely, the average BMI decreased from Q1 to Q4, while the sex distribution remained relatively even across quartiles.

**Table 1:** Characteristics of the cohort of incident VTE patients at baseline across quartiles of plasma HE4 levels.

Baseline characteristics	Plasma HE4			
	Q1	Q2	Q3	Q4
*n*	190	225	224	257
Age (y), mean ± SD	60.0 ± 12.5	66.4 ± 13.0	74.2 ± 10.8	77.5 ± 10.6
Sex (women), % (n)	46.3 (88)	50.7 (114)	54.5 (122)	51.4 (132)
BMI (kg/m ^2^ ), mean ± SD	29.8 ± 5.5	28.9 ± 4.6	28.4 ± 4.5	27.5 ± 4.5
Daily smoking, % (n)	4.7 (9)	13.3 (30)	18.8 (42)	29.2 (75)
History of CVD, % (n)	3.7 (7)	8.4 (19)	12.1 (27)	23.3 (60)

Abbreviations: BMI, body mass index; CVD, cardiovascular disease; HE4, human epididymis protein 4; SD, standard deviation; Q, quartile; VTE, venous thromboembolism.

Of the 896 participants included in the study, 116 experienced a recurrent event. The clinical characteristics at diagnosis for both incident and recurrent VTE are shown in Table 2 . The mean age at incident VTE was 70.2 years. More incident VTEs were PEs than DVTs (57.8% vs. 42.2%), while there were more DVTs than PEs (53.4% vs. 46.6%) at recurrence. More incident VTEs were provoked (57.0%), while more recurrent VTEs were unprovoked (59.5%). The proportions of cancer-associated incident and recurrent events were very similar, 20.8 and 20.7%, respectively.

**Table 2:** Clinical characteristics of the incident and recurrent VTE events at diagnosis.

Clinical characteristics	Incident VTE ( *n* = 896)	Recurrent VTE ( *n* = 116)
Age (y), mean ± SD	70.2 ± 13.5	70.8 ± 13.0
DVT, % ( *n* )	42.2 (378)	53.4 (62)
PE, % ( *n* )	57.8 (518)	46.6 (54)
Provoked VTE ^a^ , % ( *n* )	57.0 (511)	40.5 (47)
Unprovoked VTE, % ( *n* )	43.0 (385)	59.5 (69)
CT, % ( *n* )	20.8 (186)	20.7 (24)

Abbreviations: CT, cancer-associated thrombosis; DVT, deep vein thrombosis; PE, pulmonary embolism; SD, standard deviation; VTE, venous thromboembolism.

^a^ Provoked events include CT.

The baseline characteristics (measured at inclusion in the source cohort) for those who had a recurrent VTE ( *n* = 116) and those who did not ( *n* = 780) are shown in **Supplementary Table S1** . The mean age of those who suffered a recurrent VTE was lower than those who did not, while the mean BMI was higher. The percentage of women, daily smokers, and those with a history of CVD was lower amongst participants with a recurrent VTE.

The 5-year cumulative incidence of VTE recurrence according to quartiles of plasma HE4 levels is shown in Fig. 2 . The cumulative incidence curve showed no clear relationship between quartiles of increasing HE4 levels and risk of recurrent VTE.

**Fig. 2:**
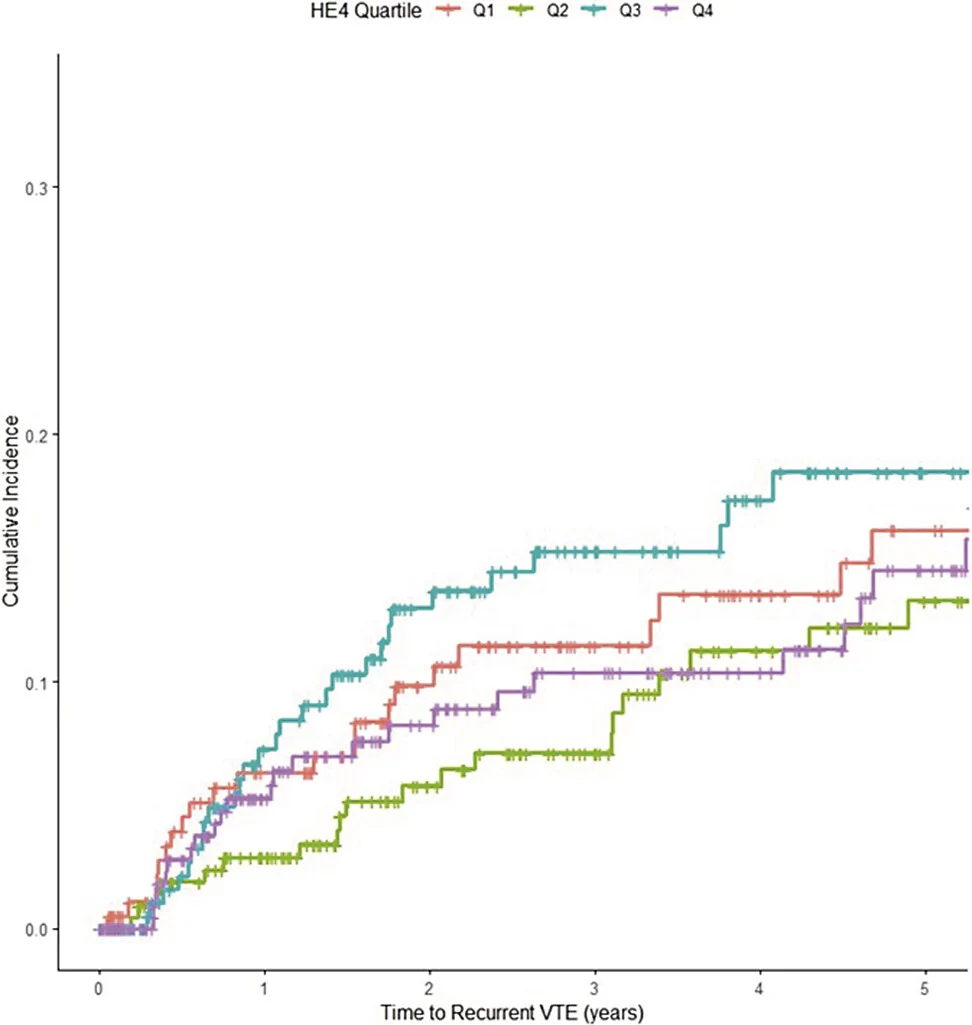
Cumulative incidence curve for recurrent VTE across quartiles of plasma HE4 levels. HE4, human epididymis protein 4; Q, quartile; VTE, venous thromboembolism.

The IRs and HRs with corresponding 95% CIs for recurrent VTE according to incident overall and subtypes of VTE and quartiles of plasma HE4 levels are shown in Table 3 . The IR of VTE recurrence among all incident VTEs was 3.31 (95% CI: 2.21–4.75) in the highest quartile (Q4) and 3.68 (95% CI: 2.43–5.36) per 100 PY in the lowest quartile (Q1). Individuals with elevated plasma HE4 levels (Q4) had essentially similar risk of recurrent VTE compared with those with low HE4 levels (Q1; HR: 0.82, 95% CI: 0.46–1.49) in the analysis adjusted for age and sex (model 1) and in the analysis additionally adjusted for BMI (model 2). Coherently, the cubic spline plot showed no association between HE4 modeled as a continuous variable and risk of recurrence after overall VTE ( **Supplementary Fig. S1** ).

**Table 3:** IRs and HRs with 95% CIs for overall VTE recurrence according to quartiles of plasma HE4 levels measured at study inclusion and based on type of incident event.

Incident event	Plasma HE4	Events	PY	IR (95% CI) ^b^	Model 1; HR (95% CI)	Model 2; HR (95% CI)
Overall VTE	Q1	27	732.9	3.68 (2.43–5.36)	1 (reference)	1 (reference)
	Q2	29	918.9	3.16 (2.11–4.53)	0.83 (0.49–1.42)	0.84 (0.49–1.43)
	Q3	31	762.8	4.06 (2.76–5.77)	1.02 (0.58–1.81)	1.05 (0.59–1.88)
	Q4	29	877.3	3.31 (2.21–4.75)	0.82 (0.46–1.49)	0.83 (0.45–1.53)
DVT	Q1	17	349.7	4.86 (2.83–7.78)	1 (reference)	1 (reference)
	Q2	12	412.7	2.91 (1.50–5.08)	0.61 (0.29–1.30)	0.62 (0.29–1.32)
	Q3	15	306.1	4.90 (2.74–8.08)	1.10 (0.50–2.42)	1.13 (0.51–2.50)
	Q4	14	401.5	3.49 (1.91–5.85)	0.76 (0.34–1.71)	0.73 (0.32–1.67)
PE	Q1	10	383.2	2.61 (1.25–4.80)	1 (reference)	1 (reference)
	Q2	17	506.2	3.36 (1.96–5.38)	1.15 (0.52–2.55)	1.14 (0.51–2.53)
	Q3	16	456.7	3.50 (2.00–5.69)	1.05 (0.44–2.47)	1.07 (0.45–2.56)
	Q4	15	475.8	3.15 (1.76–5.20)	0.91 (0.37–2.22)	0.97 (0.39–2.42)
Provoked VTE ^a^	Q1	15	425.4	3.53 (1.97–5.82)	1 (reference)	1 (reference)
	Q2	13	456.9	2.85 (1.51–4.87)	0.78 (0.37–1.65)	0.82 (0.38–1.76)
	Q3	17	400.7	4.24 (2.47–6.79)	1.15 (0.52–2.52)	1.26 (0.56–2.82)
	Q4	12	445.9	2.69 (1.39–4.70)	0.71 (0.31–1.66)	0.78 (0.32–1.90)
Unprovoked VTE	Q1	12	307.5	3.90 (2.02–6.82)	1 (reference)	1 (reference)
	Q2	16	461.9	3.46 (1.98–5.62)	0.90 (0.42–1.93)	0.87 (0.40–1.88)
	Q3	14	362.1	3.87 (2.11–6.49)	0.91 (0.39–2.14)	0.89 (0.38–2.08)
	Q4	17	431.4	3.94 (2.30–6.31)	0.95 (0.41–2.23)	0.92 (0.39–2.17)

Abbreviations: BMI, body mass index; CT, cancer-associated thrombosis; CI, confidence interval; DVT, deep vein thrombosis; HE4, human epididymis protein 4; HR, hazard ratio; IR, incidence rate; PE, pulmonary embolism; PY, person-years; Q, quartile; VTE, venous thromboembolism.

Note: Model 1: adjusted for age and sex. Model 2: adjusted for model 1 plus BMI.

^a^ Provoked events include cancer-associated VTE.

^b^ Incidence rate per 100 PY.

As the duration of anticoagulant treatment after incident VTE affects the recurrence risk, 41 and we had no registration of actual anticoagulant treatment duration after incident VTE in our cohort, we performed a sensitivity analysis with start of follow-up 3 and 6 months following the incident VTE event ( **Supplementary Table S2** ). The IR in the analysis where the follow-up period began after the first 3 months following the incident VTE diagnosis was 3.32 (95% CI: 2.22–4.77) in Q4 and 3.42 (95% CI: 2.21–5.05) per 100 PY in Q1. In the analysis where the follow-up period began after the first 6 months following the VTE diagnosis, the IR was 2.65 (95% CI: 1.68–3.98) in Q4 and 2.75 (95% CI: 1.68–4.25) per 100 PY in Q1. The corresponding model 2 HRs were 0.93 (95% CI: 0.50–1.68) and 1.03 (95% CI: 0.52–2.04) when follow-up started after 3 and 6 months, respectively ( **Supplementary Table S2** ).

The varying time lapse between blood sampling and incident VTE could introduce nondifferential misclassification of the exposure (HE4 levels) and lead to underestimation of the true association between HE4 and recurrence. **Supplementary Table S3** displays the IRs and HRs with corresponding 95% CIs for recurrent VTE according to quartiles of plasma HE4 levels in the analysis adjusted for the time lapse between blood sampling and incident VTE (sensitivity analysis). The results remained essentially unchanged for overall (HR: 0.84, 95% CI: 0.45–1.56), as well as for all the subgroup analyses restricted to the types of index VTE.

## Discussion

Results from unsupervised plasma proteome studies 42 43 and a case-cohort study 35 with adjustments for major confounders do indicate that elevated plasma HE4 levels are associated with risk of future incident VTE. In this study, we sought to investigate whether plasma HE4 levels measured prior to the incident VTE were associated with risk of recurrent VTE in a cohort of VTE patients recruited from the general population. We found no significant association between quartiles of plasma HE4 levels and the cumulative incidence or HR for recurrent VTE for the entire patient cohort. Similarly, no associations were observed in analyses stratified by the type of VTE (DVT or PE) or by provoked and unprovoked VTE. Our findings suggest that inherent plasma HE4 levels are not a candidate predictive biomarker for VTE recurrence.

To our knowledge, this is the first study to investigate the association between inherent plasma HE4 levels and VTE recurrence. The rationale for investigating the association between HE4 levels and risk of VTE recurrence originated from studies 35 42 43 consistently reporting a positive relationship between HE4 levels and risk of future incident VTE. In the study by Cathro et al, individuals with plasma HE4 levels in the highest quartile (Q4) had almost threefold (HR: 2.75, 95% CI: 1.63–4.63) higher risk of future incident VTE compared with those with the lowest HE4 levels (Q1) after adjusting for putative major confounding factors. 35 In the present study, we found no association between inherent plasma HE4 levels and risk of VTE recurrence, either for overall incident VTE or for subgroups of incident VTE (DVT, PE, provoked, and unprovoked).

The apparent discrepancy of plasma HE4 as a risk biomarker for incident and recurrent VTE may be attributed to “index event bias” or “collider bias,” which occurs because recurrence studies selectively include individuals who have already experienced an incident VTE. 44 45 Thus, the population of interest consists of individuals who possess diverse risk factors (e.g., genetic factors, obesity, and surgery) that contributed to the incident VTE event, which makes it difficult for one risk factor for incident VTE (e.g., HE4) to stand out amongst the others for recurrent VTE. 44 45 This aligns with the “recurrence paradox,” a phenomenon describing that risk factors for recurrence often differ from those for incident events (e.g., factor V Leiden and prothrombin G20210A). 46 47 Although HE4 has consistently been shown to be associated with incident VTE, HE4 levels measured prior to the incident VTE did not emerge as a risk biomarker of recurrence when compared with other VTE risk factors (e.g., older age, obesity, and genetic risk factors) accumulated in patients with incident VTE.

The varying time lapse between blood sampling for HE4 measurement and the incident VTE event for individuals in the cohort might influence the recurrence risk. In the present study, plasma samples for HE4 measurements were collected at inclusion in HUNT3 (2006–2008), and thereby at various time intervals (median: 7.0 years, range: 0.04–13.2 years) prior to the incident VTE event (rather than at the acute thrombotic event). Even though the same quartile-based cut-off values were used for assessment of recurrence risk and risk of incident VTE, fluctuation of plasma HE4 levels during this time period due to changes in environmental factors (e.g., age, BMI, smoking status, and kidney function) 32 48 49 and presence of co-morbidities (e.g., cancer, inflammatory diseases, and CVDs) 23 27 30 31 50 would lead to nondifferential misclassification of the plasma HE4 levels with subsequent underestimation of the true association between inherent HE4 levels and risk of recurrent VTE. 51 However, weekly sampling and measurement of HE4 for 5 weeks in healthy individuals displayed low within-subject biological variability (5.1–10.5%). 52 Furthermore, adjustment for the time lapse from blood sampling to the incident VTE did not affect the risk estimates for the association between inherent HE4 levels and risk of recurrent VTE (sensitivity analyses). Thus, it is less likely that variability in the time lapse between blood sampling and incident VTE would influence the association between inherent HE4 levels and recurrent VTE. An alternative approach would be to investigate whether HE4 levels measured at the acute VTE event would be associated with risk of recurrent VTE. This approach remains to be investigated.

Strengths of our study include the high participation rate in HUNT3, inclusion of all symptomatic and objectively confirmed incident and recurrent VTE cases during the follow-up period, and storage of plasma samples in a high-quality biobank. Additionally, protein quantification was conducted using the Somascan 7k platform, which is independently verified to have a high sensitivity and low variability, 53 with a CV of 5.3% for HE4. 40 This study also has some limitations. First, as the time lapse from blood sampling to incident VTE varied in the cohort of participants with incident VTE, repeated measurements of HE4 levels during follow-up could improve accuracy and limit misclassification of HE4 levels. Notably, sensitivity analysis adjusting for the time lapse between blood sampling and incident VTE had essentially no impact on the results. Although the long-term intra-individual variation in HE4 levels has not been assessed, within-subject biological variation of plasma HE4 levels only varied from 5.1% to 10.5% over a 5-week period in a study of healthy participants. 52 Nonetheless, intraindividual fluctuations over a long follow-up period could have led to nondifferential misclassification of HE4 levels and thereby underestimation of the true association between HE4 and VTE recurrence. The duration of anticoagulant treatment is known to impact the recurrence risk. 17 41 As we did not have information regarding duration of anticoagulant treatment after incident VTE, we performed sensitivity analyses in which follow-up was started 3 and 6 months 17 following the incident VTE. These analyses showed similar risk estimates as the main analysis. Our study population is predominantly Caucasian, which limits the generalizability of our findings to other ethnicities.

## Conclusion

In conclusion, plasma HE4 levels measured prior to incident VTE were not associated with risk of recurrence in a cohort of participants with incident VTE. Our results suggest that inherent plasma HE4 levels are not useful as a biomarker for risk of recurrent VTE.

**What Is Known on This Topic?**

- Plasma human epididymis protein 4 (HE4) is associated with increased risk of first lifetime venous thromboembolism (VTE).
- Whether HE4 is associated with VTE recurrence has not been addressed.

**What Does This Paper Add?**

- We have investigated the association between HE4 and risk of VTE recurrence in 896 individuals with first-lifetime VTE.
- Our findings indicate that plasma HE4 is not a useful risk biomarker for recurrent VTE.

## Data Availability

Access to data from the Trøndelag Health study can be obtained by application to the HUNT administration ( https://www.ntnu.edu/hunt/data ).

## Supplementary Material

Ergänzendes Material Supplementary Material
