# Supplementary material for: Plasma Level of Human Epididymis Protein 4 and Risk of Recurrence after First Venous Thromboembolism: The HUNT Study
Source: TH Open. 2026 Sep 30;10:a29612256. doi: 10.1055/a-2961-2256 (PMC13643896; doi:10.1055/a-2961-2256)
Supplement: Supplementary file 1 [file 10-1055-a-2961-2256_29673437.pdf]

Supplementary Material to "Plasma level of human epididymis protein 4 and risk of recurrence after first venous thromboembolism – The HUNT Study"

Supplementary Table S1. Baseline characteristics of the entire cohort.

Baseline Characteristics	Recurrent VTE (n=116)	No Recurrent VTE (n=780)
Age (years), mean ± SD	68.1 ± 13.4	70.5 ± 13.4
Sex (women), % (n)	49.1 (57)	51.2 (399)
BMI (kg/m^2), mean ± SD	29.3 ± 5.5	28.5 ± 4.7
Smoking, % (n)	15.5 (18)	17.7 (138)
History of CVD, % (n)	7.8 (9)	13.3 (104)

BMI, body mass index; CVD, cardiovascular disease; SD, standard deviation; VTE, venous thromboembolism.

TH Open | DOI 10.1055/a-2961-2256 | © The Author(s).

Supplementary Table S2. Sensitivity analysis where the follow-up time begins after the first 3 and 6 months following incident VTE diagnosis. HRs and 95% CIs for overall VTE recurrence according to quartiles of plasma HE4 levels measured at study inclusion and based on all incident VTEs.

Timing of recurrent VTE	Plasma HE4	Events	PY	IR (95% CI)*	Model 1 HR (95% CI)	Model 2 HR (95% CI)
> 3 months after incident VTE	Q1	25	731.4	3.42 (2.21-5.05)	1 (reference)	1 (reference)
	Q2	27	916.7	2.95 (1.94-4.29)	0.84 (0.48-1.47)	0.85 (0.49-1.48)
	Q3	31	759.9	4.08 (2.77-5.79)	1.14 (0.63-2.04)	1.17 (0.64-2.11)
	Q4	29	873.5	3.32 (2.22-4.77)	0.92 (0.50-1.68)	0.93 (0.50-1.72)
> 6 months after incident VTE	Q1	20	727.3	2.75 (1.68-4.25)	1 (reference)	1 (reference)
	Q2	25	914.5	2.73 (1.77-4.04)	0.99 (0.55-1.81)	1.01 (0.55-1.84)
	Q3	27	754.1	3.58 (2.36-5.21)	1.32 (0.69-2.51)	1.37 (0.71-2.63)
	Q4	23	867.6	2.65 (1.68-3.98)	0.97 (0.50-1.90)	1.03 (0.52-2.04)

*Incidence rate per 100 PY.

Model 1: adjusted for age and sex.

Model 2: adjusted for model 1 plus BMI.

CI, confidence interval; HE4, human epididymis protein 4; HR, hazard ratio; IR, incidence rate; PY, person-years; Q, quartile; VTE, venous thromboembolism.

TH Open | DOI 10.1055/a-2961-2256 | © The Author(s).

Supplementary Table S3. Sensitivity analysis adjusting for timelapse between blood sampling and incident VTE as a covariate. IRs and HRs with 95% CIs for overall VTE recurrence according to quartiles of plasma HE4 levels measured at study inclusion and based on type of incident event.

Incident event	Plasma HE4	Events	PY	IR (95% CI)*	Model 2 HR (95% CI)	Model 2 + time HR (95% CI)
Overall VTE	Q1	27	732.88	3.68 (2.43-5.36)	1 (reference)	1 (reference)
	Q2	29	918.87	3.16 (2.11-4.53)	0.84 (0.49-1.43)	0.84 (0.49-1.44)
	Q3	31	762.80	4.06 (2.76-5.77)	1.05 (0.59-1.88)	1.06 (0.59-1.90)
	Q4	29	877.30	3.31 (2.21-4.75)	0.83 (0.45-1.53)	0.84 (0.45-1.56)
DVT	Q1	17	349.65	4.86 (2.83-7.78)	1 (reference)	1 (reference)
	Q2	12	412.72	2.91 (1.50-5.08)	0.62 (0.29-1.32)	0.62 (0.29-1.33)
	Q3	15	306.06	4.90 (2.74-8.08)	1.13 (0.51-2.50)	1.15 (0.52-2.57)
	Q4	14	401.46	3.49 (1.91-5.85)	0.73 (0.32-1.67)	0.77 (0.33-1.77)
PE	Q1	10	383.23	2.61 (1.25-4.80)	1 (reference)	1 (reference)
	Q2	17	506.15	3.36 (1.96-5.38)	1.14 (0.51-2.53)	1.12 (0.50-2.50)
	Q3	16	456.74	3.50 (2.00-5.69)	1.07 (0.45-2.56)	1.05 (0.44-2.53)
	Q4	15	475.83	3.15 (1.76-5.20)	0.97 (0.39-2.42)	0.93 (0.36-2.38)
Provoked VTE	Q1	15	425.37	3.53 (1.97-5.82)	1 (reference)	1 (reference)
	Q2	13	456.94	2.85 (1.51-4.87)	0.82 (0.38-1.76)	0.82 (0.38-1.76)
	Q3	17	400.71	4.24 (2.47-6.79)	1.26 (0.56-2.82)	1.25 (0.56-2.81)
	Q4	12	445.94	2.69 (1.39-4.70)	0.78 (0.32-1.90)	0.77 (0.31-1.90)
Unprovoked VTE	Q1	12	307.50	3.90 (2.02-6.82)	1 (reference)	1 (reference)
	Q2	16	461.93	3.46 (1.98-5.62)	0.87 (0.40-1.88)	0.88 (0.41-1.92)
	Q3	14	362.08	3.87 (2.11-6.49)	0.89 (0.38-2.08)	0.90 (0.38-2.14)
	Q4	17	431.36	3.94 (2.30-6.31)	0.92 (0.39-2.17)	0.96 (0.40-2.32)

*Incidence rate per 100 PY.

Model 2: adjusted for age, sex, and BMI.

Model 2 + time: adjusted for model 2 plus time.

BMI, body mass index; CI, confidence interval; DVT, deep vein thrombosis; HE4, human epididymis protein 4; HR, hazard ratio; IR, incidence rate; PE, pulmonary embolism; PY, person-years; Q, quartile; VTE, venous thromboembolism.

TH Open | DOI 10.1055/a-2961-2256 | © The Author(s).

Supplementary figure S1. Cubic spline plot showing the hazard ratio (HR) with 95% confidence interval (95% CI) of venous thromboembolism (VTE) recurrence according to increasing plasma levels of human epididymis protein 4 (HE4).

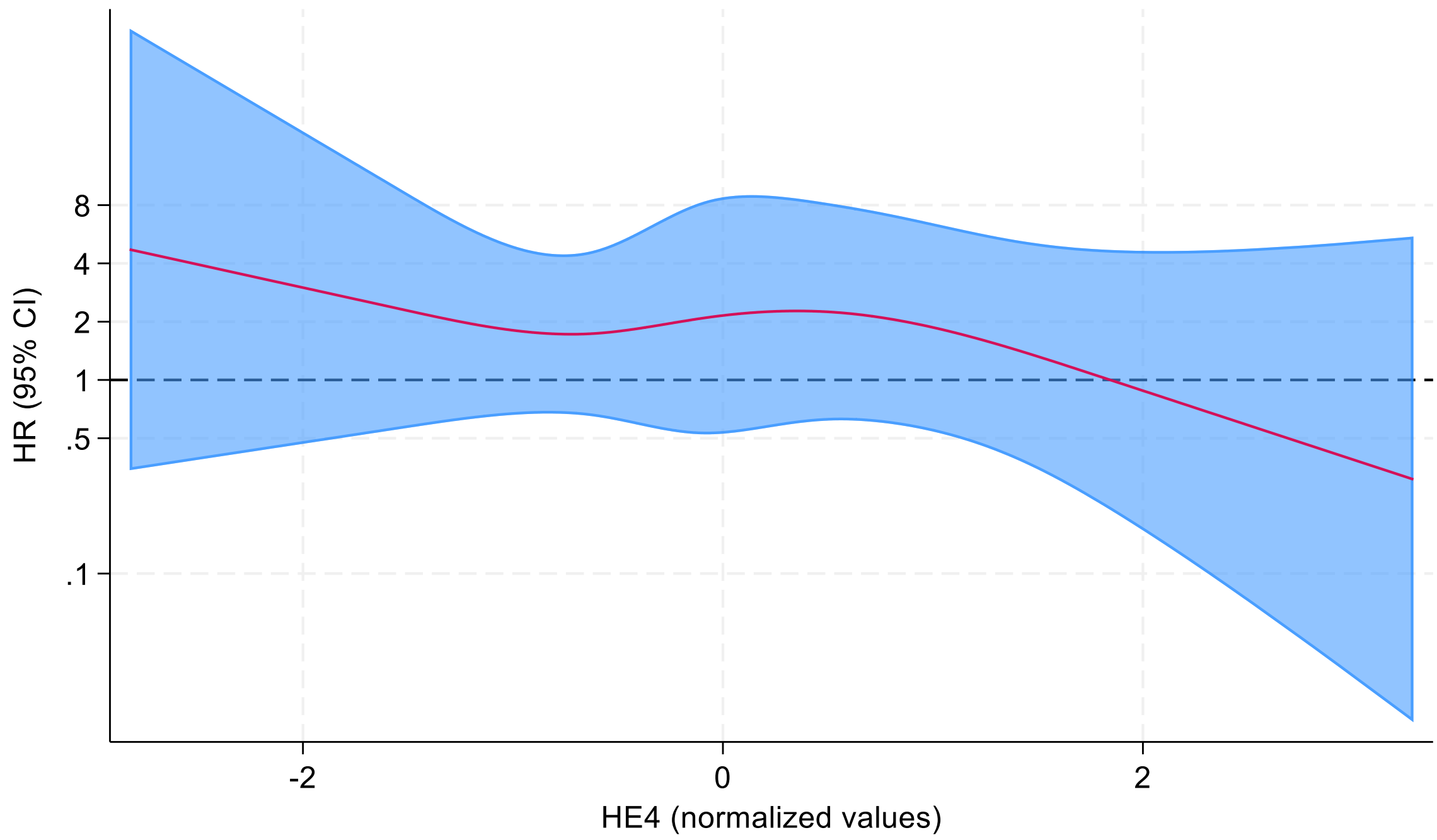

Normalized values: HE4 was log2 transformed and standardized to yield a mean of 0 and standard deviation of 1 before entering the analyses. Red line: HR, Shaded blue area: 95% CI.

TH Open | DOI 10.1055/a-2961-2256 | © The Author(s).
